# A 20-year research trend analysis of the artificial intelligence on scoliosis using bibliometric methods

**DOI:** 10.3389/fped.2025.1531827

**Published:** 2025-08-13

**Authors:** Bin Zheng, Zhenqi Zhu, Yan Liang, Chen Guo, Haiying Liu

**Affiliations:** Spine Surgery, Peking University People’s Hospital, Beijing, China

**Keywords:** artificial intelligence, scoliosis, adolescent idiopathic scoliosis, deep learning, machine learning, bibliometrics

## Abstract

**Background:**

This bibliometric analysis aimed to map the knowledge network of artificial intelligence in scoliosis

**Methods:**

Studies on artificial intelligence published from January 2003 to December 2024 are retrieved from Web of Science Core Collection (WoSCC). The contributions of countries, institutions, authors, and journals are identified using VOSviewer, Online Analysis Platform of Literature Metrology (http://biblimetric.com) and Microsoft Excel. Tendencies, hotspots and knowledge networks are analyzed and visualized using VOS-viewer and CiteSpace.

**Results:**

718 publications are included in the final analysis. The leading country in this field is China. Royal Hospital for Sick Children featured the highest number of publications among all institutions and National University of Singapore featured the highest citations of publications. Co-citation cluster labels revealed characteristics of three main clusters: (1) Image process and classification of scoliosis, (2) AI application in surgical treatment of scoliosis, (3) predict postoperative complications and scoliosis development. Keyword burst detection indicated that machine learning and deep learning are the newly emerging research hot spots.

**Conclusion:**

This study compiled 718 publications covering AI in scoliosis and showed that the direction of these studies is likely in transition from cerebral palsy to machine learning and deep learning. It provides guidance for further research and clinical applications on AI application in scoliosis.

## 1 Introduction

The diagnosis and treatment of scoliosis is major focus in spine surgery. Scoliosis is a complex three-dimensional spinal deformity, classified into various types based on etiology and age of onset, including idiopathic, neuromuscular, congenital, and degenerative scoliosis. Adolescent idiopathic scoliosis (AIS) is the most common structural spinal deformities in adolescents, with a global prevalence of 0.47%–5.2% (1). AIS is a complex three-dimensional spinal deformity with an unclear etiology. Severe AIS can lead to cardiopulmonary dysfunction, and corrective surgery is often necessary when the Cobb angle exceeds 40 degrees. Additionally, with the aging population, the prevalence of ADS is also increasing (2, 3). Compared to AIS, the pathophysiological changes in ADS are more complex, and its clinical manifestations are more varied. The intricate structural changes in ADS present greater challenges for surgical intervention (4–6). Spine deformities not only affect spinal balance, causing compensation between the spine and the trunk, but in severe cases, they can also lead to cardiopulmonary dysfunction and even spinal cord damage (7). Congenital scoliosis refers to scoliosis caused by abnormal vertebral development due to genetic, environmental, or other factors during embryonic development. It typically becomes apparent from early childhood to adolescence. Congenital scoliosis (CS) can be classified into three categories based on the mechanism of vertebral deformity: segmentation defects, formation failures, and mixed types.

Artificial Intelligence (AI) technology, which simulates human intelligence, includes machine learning, computer vision, and natural language processing (8, 9). AI has shown great potential in spine surgery research and clinical applications (10). Numerous studies have confirmed the value of AI in automatic measurement of the Cobb angle, creation of three-dimensional models of AIS from biplanar radiographs, automatic screening of AIS, classification of scoliosis deformities, and fully automated analysis of spinal parameters (11, 12). Furthermore, in the diagnosis and treatment of spinal deformities, AI offers significant advantages in interpreting x-rays, CT scans, and MRI images, recognizing spine segments, planning the surgery planning in complex spinal surgeries, precisely inserting screws during surgery, performing finer osteotomies, and assisting in postoperative rehabilitation of severe cases (13). These advancements have significantly improved the efficiency and accuracy of scoliosis diagnosis and treatment, allowing AI to assist doctors in more precisely addressing complex spinal cases, optimizing surgical outcomes, and accelerating patient recovery. This integration of AI not only transforms clinical practice but also provides a vast potential for the future of spine surgery.

Exploring the recent research status and development trends of AI in scoliosis is beneficial for gaining a deeper understanding of AI's role in spine scoliosis, identifying research hotspots, and setting future research directions. Bibliometric analysis, a relatively new method of literature research, can qualitatively and quantitatively evaluate the development trends of research activities based on information provided by literature databases. This approach helps grasp the development of a particular field and provides a way to compare contributions at different levels. However, there are currently no studies or reports on the quantity and quality of literature research on AI in scoliosis. This article aims to summarize the global research status of AI application in scoliosis from a bibliometric and visualization analysis perspective and predict its development trends.

## 2 Materials and methods

### 2.1 Data source and search strategy

Since 2003, artificial intelligence technology has undergone rapid development and transformation. During this period, AI has progressed from a relatively preliminary stage toward maturity, with algorithms continually optimized and applications expanding across various fields. The literature search is performed on the WoSCC website to identify publications indexed between 1 January 2003, and 31 December 2024. The specific search formula is as follows: Topic (TS) = (artificial intelligence OR AI OR deep learning OR intelligent OR robot t OR language model OR Convolutional Neural Network) AND (scoliosis). Only studies published in English are included. To avoid bias, two independent investigators (Bin Zheng and Zhenqi Zhu) performed the literature search and filtering and a senior researcher (Haiying Liu) resolved any discrepancies in findings between these investigators.

### 2.2 Inclusion and exclusion criteria

This analysis included review and articles on the AI in scoliosis in the WoSCC database between 1st January 2003, and 31st December 2024. The exclusion criteria are (1) unpublished papers, (2) articles requiring manual research, and (3) articles written in languages other than English. 718 are finally included in the analyses.

### 2.3 Data extraction and bibliometric analysis

The extracted bibliometric parameters included journal names, publication times, titles, countries/regions, institutions, authors, keywords, references, and citations. Journal impact factors (IFs) are collected from the most recent Journal Citation Reports (2023). In addition, VOSviewer (version 1.6.20), CiteSpace (version 6.3 R1) and Microsoft Excel 2019 are used to perform the bibliometric analysis and visualization.

Microsoft Excel is used for the time and contribution analyses. For analysis of different countries' publication trends, the WoSCC data is converted to UTF-8 format and imported into the Online Analysis Platform of Bibliometrics (http://bibliometric.com/) choosing the “total literature analysis” option. For intercountry/regional analysis, we chose the “partnership analysis” option.

VOSviewer is used to visualize keyword co-occurrence network. CiteSpace is used to visualize the keyword and reference burst figures and reference citation network. The choice of VOSviewer is due to its clear and intuitive visualization advantages and robust clustering capabilities, which help researchers quickly identify core topics, authors, and institutions, and clarify research topic directions. CiteSpace, on the other hand, is selected for its expertise in constructing knowledge maps, which can reveal emerging trends by analyzing citation relationships and keyword co-occurrence to identify key nodes and development trends. It also allows time-series analysis to display dynamic changes over time. VOSviewer and CiteSpace complement each other, with the former focusing on visualization and clustering analysis and the latter on knowledge mapping and dynamic analysis. Together, they provide a comprehensive and in-depth understanding of AI applications in scoliosis research and enhance the reliability and accuracy of the research through multi-angle verification.

## 3 Results

### 3.1 Trend analysis of published papers

**Figure 1:**
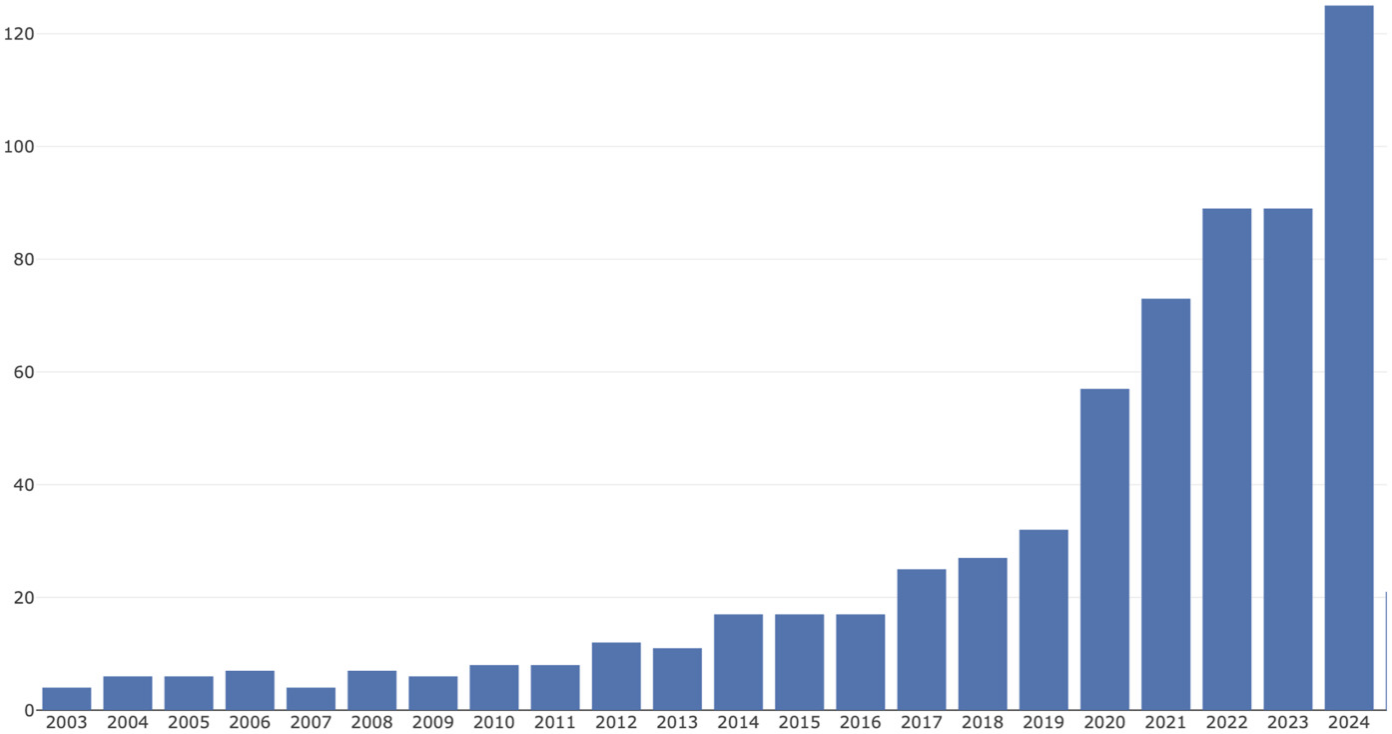
Annual trend in publications on AI applications in scoliosis research from 2003 to 2024.

Between 1st January, 2003, and 31st December, 2024, a total of 718 publications on AI in scoliosis are identified, as shown in Figure 1. Overall, there is a significant upward trend in the number of publications over this 21-year period. This timeline can be divided into three stages.

**I. Early Years (2003–2011):** The number of publications remained relatively stable, fluctuating between 4 and 8 per year. **II. Mid-period growth (2012–2018):** A steady increase began, with publications rising from about 12 per year to over 50.; and **Rapid growth (2019–2024):** A sharp acceleration occurred, with the number of publications more than doubling from about 54 in 2019 to a peak of nearly 133 in 2024. This trend suggests that research on AI in scoliosis has become an increasingly popular focus area in recent years.

### 3.2 Analysis of global leaders and cooperation in AI research on scoliosis by country/region

**Figure 2:**
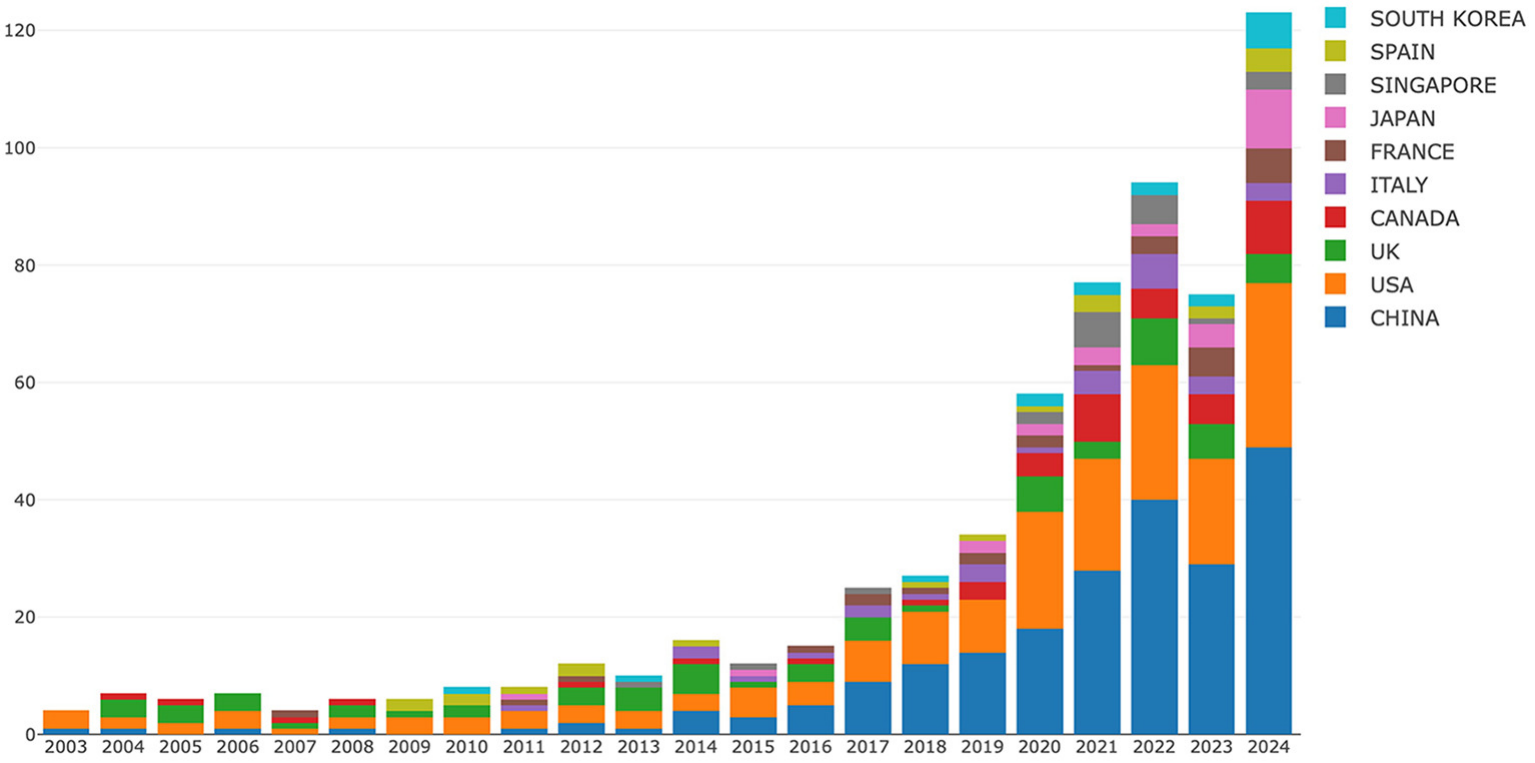
Annual publications on AI applications in scoliosis research from the top 10 countries/regions (2003–2024). This stacked bar chart illustrates the distribution of publications across leading countries, with notable contributions from China, the USA, and the UK, particularly from 2019 onwards. Each color represents a different country, with China (blue) and the USA (orange) showing a significant rise in publication volume.

In order to find out which countries/regions are leading in research in this field, further analysis of publications in different countries and regions is conducted using the Online Analysis Platform of Bibliometrics (http://bibliometric.com/), shown in Figure 2. China (shown in blue) experiences significant growth, especially from 2017 onwards, becoming the largest contributor in recent years. USA (in orange) also shows considerable growth over time, particularly in the middle years of the period shown. Other countries like Canada, Italy, Singapore contribute smaller but still notable quantities.

**Figure 3:**
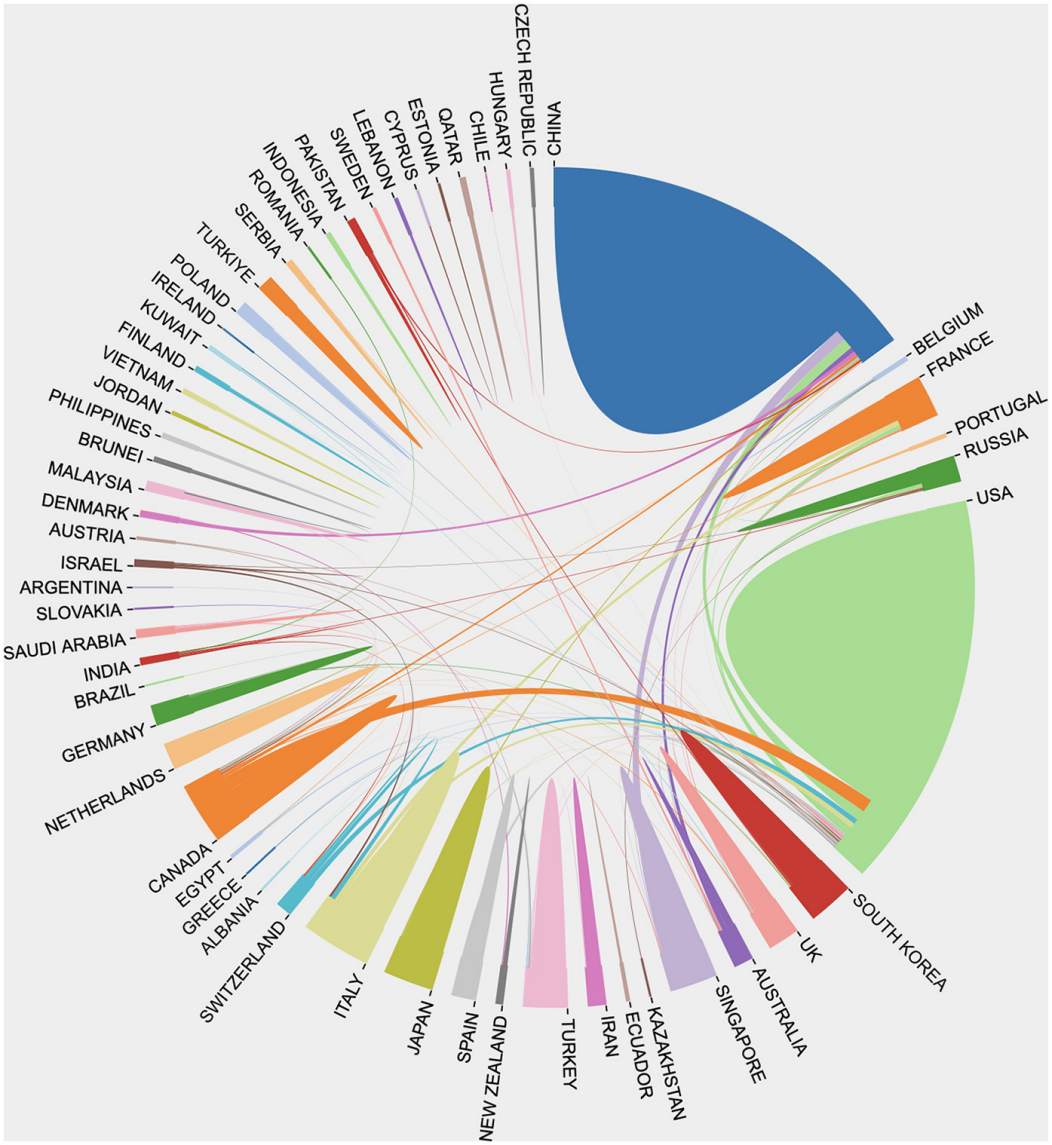
International collaboration network in AI applications for scoliosis research, showing the interconnectedness between countries and regions. Each segment represents a country, with links indicating collaborative publications between them. Major contributors, such as China (blue), the USA (green), and the UK (orange), display extensive connections, highlighting frequent collaborations with multiple countries. This diagram underscores the global, cooperative nature of AI research in scoliosis, illustrating how knowledge exchange between countries is accelerating advancements in the field.

60 countries/regions have contributed to this field, and the top ten of them are listed in Table 1. Among these, the **China** is first, with 234 publications, followed by **USA** (179 publications) and **UK** (72 publications). Regarding Total Citation, the **China** ranked first (3,773), followed by **USA** (3,543) and **Singapore** (1,512). The results underscores both the global distribution of research efforts and the key roles of USA and China. Results of intercountry/regional cooperation suggested cooperation between partnerships, especially the USA and China are the key center for cooperation, shown in Figure 3.

**Table 1:** Top 10 contribution and citation countries/regions to AI in scoliosis.

Country/region	Documents	Citation	Citation/Documents
China	234	3,773	16.12
USA	179	3,543	19.79
UK	72	1,426	19.81
Canada	46	498	10.83
Italy	28	735	26.25
France	28	412	14.71
Singapore	21	1,512	72.00
Spain	21	211	10.05
South Korea	17	147	8.65
Switzerland	16	289	18.06

### 3.3 Institutions analysis

In total, 919 institutions participated in the publication of articles on AI in scoliosis. Among the top ten productive institutions, 4 are located in the **China**, 4 in USA, 1 in UK and 1 in Singapore. **Royal Hospital for Sick Children** contributed the most publications (28 publications), followed by **Hong Kong University** (18 publications), shown in Table 2. Regarding total citation, the top institutions are National University of Singapore (total citation = 1,483) and Royal Hospital for Sick Children (total citation = 597). “Citation/documents” indicator represents the average number of citations per document. It reflects how frequently research from an intuition is cited. This indicates that the National University of Singapore publishes studies of the highest quality, representing NUS's strong research standards and greater influence.

**Table 2:** Top contribution and citation institutions to AI in scoliosis.

Institution	Country	Documents	Citation	Citation/documents
Royal Hospital for Sick Children	UK	28	597	21.32
Hong Kong University	China	18	82	4.56
Hong Kong Polytech University	China	17	121	7.12
National University of Singapore	Singapore	15	1,483	98.87
Columbia University	USA	13	149	11.46
University of California, San Francisco	USA	13	378	29.08
Nemours/AI duPont Hospital for Children	USA	12	223	18.58
Chinese Academy of Sciences	China	12	249	20.75
Hospital for Special Surgery	USA	10	164	16.40
Tsinghua University	China	10	93	9.30

### 3.4 Top prolific authors analysis

Table 3 shows the top ten cited authors. Among the authors, Athanasios I Tsirikos publishes the most papers (51 articles) with a total of 1,261 citations, making outstanding contributions in both quantity and total citations. Chengkuo Lee, despite publishing only 11 articles, achieves the highest total citations of 1,285 among individual authors, with an average citation rate per paper far exceeding others, indicating stronger research impact.

**Table 3:** Top contribution authors to AI in scoliosis.

Author	Country	Institution	Documents	Citation	Citation/document
Athanasios I Tsirikos	UK	Royal Hospital for Children and Young People	51	1,261	24.73
Chengkuo Lee	Singapore	National University of Singapore	11	1,285	116.82
Agrawal, Sunil K.	USA	Columbia University	9	87	9.67
Lou, Edmond	Canada	University of Alberta	9	47	5.22
Fabio Galbusera	Italy	IRCCS Istituto Ortopedico Galeazzi	9	351	39.00
Rui Zheng	China	Shanghai Tech University	9	64	7.11
Jaime-Castillo, Sergio	Spain	University of Granada	7	23	3.29
Cheriet, Farida	Canada	Sainte-Justine Hospital	7	104	14.86
Shah, Suken	USA	Nemours/AI duPont Hospital for Children	7	58	8.29
Teng Zhang	China	Nanjing University of Information Science & Technology	7	76	10.86

### 3.5 Journals analysis

The top ten active journals and cited journals are listed in Table 4. The impact factors are based on the Journal Citation Reports 2023. In terms of publication quantity, European Spine Journal is first, with 29 publications, followed by Spine (24 publications) and Global Spine Journal (16 publications). Regarding citations, European Spine Journal ranks first (1,056 citations), followed by Spine (595 citations). Theses indicate that the European Spine Journal leads in both publication count and citation frequency, highlighting its influence in the field. Meanwhile, Spine and Global Spine Journal also contribute significantly to the field, with notable publication quantities and citation counts.

**Table 4:** Top 10 contribution and citation journal to AI in scoliosis.

Journal	Documents	Citation	Citation/Documents^1^	IF*
European Spine Journal	29	1,056	36.41	2.6
Spine	24	595	24.79	2.6
Global Spine Journal	16	166	10.38	2.6
Journal of Pediatric Orthopaedics	14	158	11.29	1.4
Spine Deformity	13	58	4.46	1.6
Journal of Clinical Medicine	12	57	4.75	3
World Neurosurgery	12	166	13.83	1.9
IEEE Access	11	129	11.73	3.4
Bone & Joint Journal	10	253	25.30	4.9
Journal Of Spinal Disorders & Techniques	9	196	21.78	N/A

### 3.6 Top cited references analysis

Table 5 lists the top 10 most-cited papers in AI in scoliosis, with the number of citations ranging from 126–308. Article “Augmented tactile-perception and haptic-feedback rings as human-machine interfaces aiming for immersive interactions” has the highest number of citations (308), followed by “Technologies toward next generation human machine interfaces: From machine learning enhanced tactile sensing to neuromorphic sensory systems” (224 citations) and “Cobb Angle Measurement of Spine from x-Ray Images Using Convolutional Neural Network” (176 citations).

**Table 5:** Top 10 most cited article in AI on scoliosis.

Rank	Title	Author	Journal	Country	Affiliation	Year	Citation
1	Augmented tactile-perception and haptic-feedback rings as human-machine interfaces aiming for immersive interactions	Sun, Zhongda	Nature Communications	Singapore	National University of Singapore	2022	308
2	Technologies toward next generation human machine interfaces: From machine learning enhanced tactile sensing to neuromorphic sensory systems	Zhu, Minglu	Applied Physics Reviwes	Singapore	National University of Singapore	2020	224
3	Cobb Angle Measurement of Spine from x-Ray Images Using Convolutional Neural Network	Ming-Huwi Horng	Computational And Mathematical Methods In Medicine	Singapore	National University of Singapore	2020	176
4	Artificial intelligence and machine learning in spine research	Fabio Galbusera	JOR Spine	Italy	IRCCS Istituto Ortopedico Galeazzi	2019	167
5	Active self-correction and task-oriented exercises reduce spinal deformity and improve quality of life in subjects with mild adolescent idiopathic scoliosis. Results of a randomised controlled trial	Marco Monticone	European Spine Journal	Italy	IRCCS Istituto Ortopedico Galeazzi	2014	167
6	Robotic-assisted pedicle screw placement: lessons learned from the first 102 patients	Xiaobang Hu	European Spine Journal	USA	Texas Health Presbyterian Hospital Plano	2012	165
7	Low cost exoskeleton manipulator using bidirectional triboelectric sensors enhanced multiple degree of freedom sensory system	Minglu Zhu	Nature Communications	Singapore	National University of Singapore	2021	158
8	Bone-mounted miniature robotic guidance for pedicle screw and translaminar facet screw placement: Part I—Technical development and a test case result	Isador H Lieberman	Neurosurgery	USA	Cleveland Clinic	2006	130
9	Anterior vertebral body tethering for immature adolescent idiopathic scoliosis: one-year results on the first 32 patients	Amer F Samdani	European Spine Journal	USA	Shriners Hospitals for Children	2015	130
10	What Is the Learning Curve for Robotic-assisted Pedicle Screw Placement in Spine Surgery?	Xiaobang Hu	Clinical Orthopaedics And Related Research	USA	Texas Health Presbyterian Hospital Plano	2014	126

### 3.7 Keywords burst and research trend analysis

**Figure 4:**
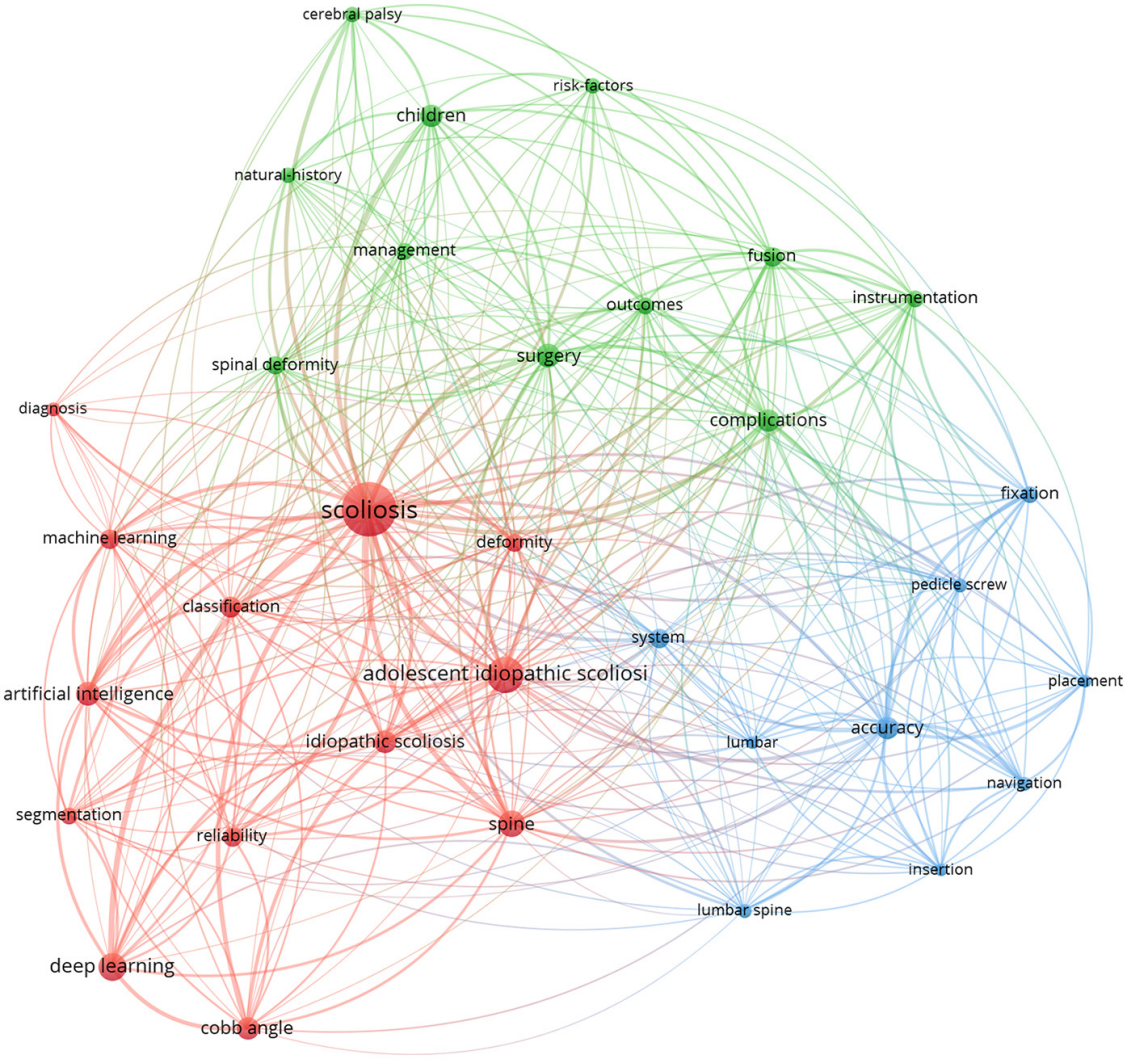
Keyword co-occurrence network in scoliosis research with AI applications. This network map visualizes frequently co-occurring keywords, clustered by thematic areas. The interconnected nodes illustrate multidisciplinary research approaches, bridging AI and clinical topics in scoliosis.

**Figure 5:**
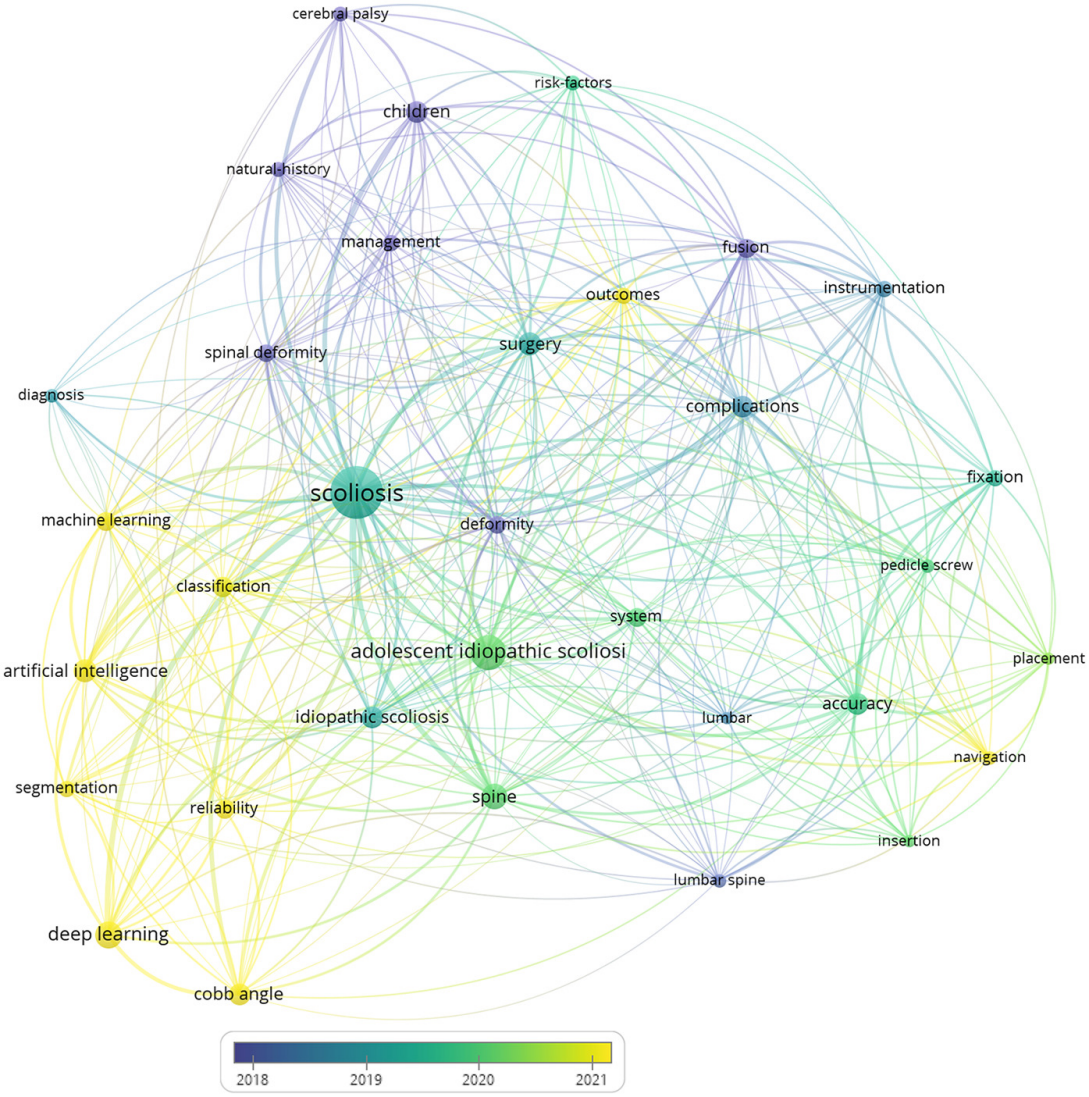
Keyword co-occurrence network in scoliosis research with AI applications, color-coded by the average publication year (2018–2021). Each node represents a keyword, with colors ranging from blue (earlier years) to yellow (more recent years), illustrating the evolution of research focus over time.

Figure 4 illustrates a visualization of keywords that co-occurred at least 15 times in AS in scoliosis. A total of 33 keywords are identified and grouped into three clusters. Cluster #1 (red) primarily focuses on the diagnosis and classification of scoliosis, as well as the use of artificial intelligence technologies (such as deep learning and machine learning) for image segmentation and angle measurement, with keywords including “scoliosis”, “adolescent idiopathic scoliosis”, “artificial intelligence”, “deep learning”, “machine learning, diagnosis”, “classification”, “segmentation”, “cobb angle”, “reliability”. Cluster #2 (blue) is primarily concerned with the technical and application in scoliosis, with keywords including “navigation systems”, “pedicle screw placement”, and “insertion accuracy”. Cluster 3 (green) addresses management of scoliosis in children., with keywords including “management,” “outcomes,”, “complications”. In addition, the keywords in Figure 5 are colored based on the average publication years. The concepts of “cerebral palsy,” “spinal deformity” appeared early (purple and green), while frontier topics including “artificial intelligence” and “deep learning,” appeared recently (yellow), which indicates

**Figure 6:**
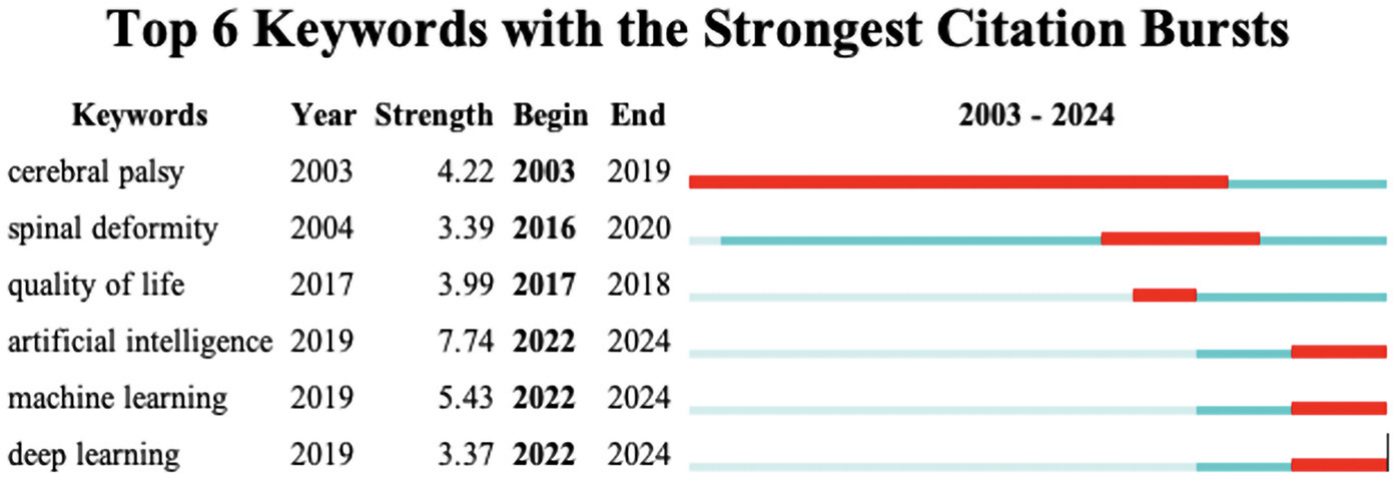
Top 6 keywords with the strongest citation bursts in scoliosis research (2003–2024). This table displays keywords that have experienced notable increases in citation frequency, signifying shifts in research focus.

Next, the CiteSpace burst module is applied to identify the research tendencies and shifts in central topics. Bursts refer to sudden increases over time. The 25 keywords with the strongest citation bursts are shown in Figure 6. Among them, the topics gradually shifted from “cerebral palsy,” “spinal deformity,” to “artificial intelligence”, “machine learning”, and “deep learning”.

## 4 Discussion

This study conducts a comprehensive bibliometric analysis to deeply explore the current status and development trends of AI applications in scoliosis research. The results reveal significant growth in this field over the past 20 years, particularly in recent years, reflecting the increasing importance and immense potential of AI in the diagnosis and treatment of scoliosis.

### 4.1 Research trends and growth patterns

From 2003–2024, the changes in the number of related research papers can be clearly divided into three stages: the early stage (2003–2011), the mid-growth stage (2012–2018), and the rapid growth stage (2019–2024). This growth pattern reflects the gradual maturation and widespread acceptance of AI technology in the medical field, particularly in scoliosis. The stable low growth in the early stage may reflect the initial exploration and proof-of-concept phase, while the steady growth in the mid-stage indicates that the field began to attract more attention and resource investment. In 2012, Hinton and his team used convolutional neural networks (AlexNet) to achieve outstanding results in the ImageNet image classification competition, surpassing traditional methods in accuracy, which highlighted the potential of deep learning. The rapid growth stage after 2019 coincides with breakthrough advancements in deep learning and other AI technologies. The maturity of deep learning technologies (such as convolutional neural networks and generative adversarial networks) has significantly improved imaging accuracy, making automated Cobb angle measurement and spinal image segmentation more precise and reliable. Additionally, the availability of cloud computing and GPUs has enabled AI to handle large-scale medical imaging. Furthermore, the abundance of data, increased international collaboration, and the growing global demand for efficient scoliosis diagnosis and treatment have created more opportunities for AI in clinical applications like imaging diagnostics, surgical navigation, and complication prediction. planning, and decision support.

### 4.2 National and institutional contributions

The dominance of China and the United States in this field is noteworthy. These two countries not only lead in the number of published articles but also rank top in total citation counts First, China has a large population base, with an AIS incidence rate of approximately 1.02%–1.2% (14, 15). This vast population and rich clinical cases provide a significant advantage, particularly in the accumulation of imaging data. Additionally, China's investment in artificial intelligence and big data infrastructure enables researchers to utilize powerful computational resources to train and optimize deep learning models, thereby improving the effectiveness and accuracy of AI application. As China's influence in AI research on scoliosis grows, its rich clinical data and research experience can be shared with other countries, fostering international multi-center collaborative research. This sharing of data and resources will facilitate the development of more widely adaptable AI systems, enhancing the applicability and adoption of AI in clinical applications worldwide. China's leading research provides a viable path for the automation of scoliosis diagnosis and treatment. AI-based diagnostic tools and surgical assistance systems can be introduced in regions with limited medical resources, improving diagnostic and treatment efficiency by reducing reliance on highly skilled personnel. Particularly in low-resource countries, this technology can help alleviate the shortage of specialized physicians, enabling more patients to access high-quality diagnosis and treatment.

However, although Singapore has a lower overall publication volume compared to China and the US, its research institution National University of Singapore have a higher average citation rate per article, indicating that their research quality and impact are not to be overlooked. This phenomenon reminds us that when evaluating research contributions, we should consider not only the quantity but also the quality and impact of the research.

It is worth noting that the results highlight the importance of international cooperation, especially between China and the United States. Such cross-national collaboration helps integrate research strengths from different countries and promotes the global sharing of knowledge and technology, thereby accelerating the development of the field.

### 4.3 Analysis of highly cited references

Analyzing highly cited references can provide insights into the important breakthroughs and most influential research directions in this field. Notably, the top-ranked highly cited papers are concentrated in areas such as tactile perception, human-machine interaction, and robot-assisted surgery. This indicates that, besides traditional diagnostic and classification applications, AI has significant potential in improving surgical precision, enhancing surgeons' operational capabilities, and improving patient experience.

The top-ranked paper explores the application of tactile perception and tactile feedback loops in human-machine interaction, which may provide inspiration for developing more intuitive and precise surgical assistance systems. The second-ranked paper focuses on machine learning-enhanced tactile perception technology, which may have important applications in improving surgical precision and surgical navigation.

The high citation rates of these research directions indicate that both the academic and clinical communities highly value the potential of AI in improving surgical precision, reducing complications, and improving patient outcomes. This also points to some possible key directions for future research.

### 4.4 Analysis of keywords burst timeline and keywords cluster

#### 4.4.1 Keyword burst and timeline

Keyword analysis reveals the evolution trends of research hotspots in this field. Early research mainly focused on fundamental clinical issues such as cerebral palsy and spinal deformities, while recent studies have more focused on the specific applications of AI, machine learning, and deep learning in the diagnosis and treatment of scoliosis. This shift reflects the influence of technological advancements on research directions and also indicates that future research may concentrate more on the clinical application and validation of AI technologies.

#### 4.4.2 Keywords cluster analysis

Keyword clustering analysis in the medical context can be categorized into three clusters.

##### 4.4.2.1 Red cluster

Accurate image process and classification of scoliosis are crucial for developing effective treatment plans. In recent years, the application of AI technology in this field has made significant progress, greatly improving the accuracy, efficiency, and consistency of diagnoses.

Image segmentation is a critical step in the diagnosis of scoliosis, traditionally performed manually by radiologists and spine surgeons. However, this method is not only time-consuming but also susceptible to subjective factors. The introduction of AI has changed this process significantly. By utilizing deep learning algorithms such as Convolutional Neural Networks (CNNs), AI systems can automatically identify and segment spinal structures in x-ray, CT, or MRI images. These algorithms, trained on large amounts of annotated data, can accurately recognize anatomical structures such as vertebrae, intervertebral discs, and nerve roots (16, 17). For example, combining x-ray images with MRI images provides more comprehensive information about the spine structure. Studies have shown that AI-assisted image segmentation is comparable to the skills of radiologists and experienced clinicians in terms of accuracy and consistency. The future is likely to show growth in the development and refinement of these capable networks (18).

**(1) Automated Cobb Angle Measurement** Although some alternative methods have been proposed (19, 20), Cobb angle is the gold standard for evaluating the degree of scoliosis. Measuring the Cobb angle is time-consuming with high interobserver and intraobserver variability. Moreover, the selection of end-vertebrae often varies, which is a major source of error. Manually selecting the end vertebrae can result in an error of up to 11.8° (21, 22). The use of AI to measure the Cobb angle is currently one of the most extensively researched methods.AI can automatically identify the most inclined upper and lower vertebrae, eliminating errors caused by manual selection. Through digital image processing techniques, AI can accurately calculate the Cobb angle. Wang used a deep learning model based on MMRotate to automatically label individual vertebrae and measure the Cobb angle. The model is trained on 227 images and validated on 70 images. Results showed an average absolute error of 1.97° with a standard deviation of 1.57°, and 95.9% of the angles had an absolute error less than 5°. The intraclass correlation coefficient is 0.981, indicating excellent reliability (23). Horng et al. used a convolutional neural networkto automatically label individual vertebrae and measure the Cobb angle. The mean absolute difference and standard deviation between the automatic and two sets of manual measurements are 3.0 ± 2.0° and 2.5 ± 1.7° (24). The automatic spinal curvature assessment deep learning model established by Pan et al. is centered on two R-CNNs. The model and observer's ICC is 0.94, with a mean absolute error of 10° (25). Implementing automated imaging measurements can not only reduce the workload of clinicians but also minimize errors caused by different measurement personnel, thereby improving the reliability of measurement parameters. However, most of the aforementioned models have limited generalization capabilities and need to be validated with multicenter data in the future.

(**2**) **Scoliosis Classification** Besides measurement of Cobb angles, accurate Lenke classification system plays a significant role in determining the treatment planning. Phan use self-organizing maps (SOM) to develop AI scoliosis system. This system highlights the tendency of surgeons to follow Lenke classification principles for similar curves. Lenke classification principles are followed in 82% of the nodes on the SOM (26). Lu developed a scoliosis diagnosis system based on the principle of oscillogram. In this system, the calculation time for each patient's data is 0.2 s, compared to 23.6 min required by each surgeon for measurements. The AI system demonstrated high accuracy in identifying Lenke classifications, even surpassing the judgment of junior spine surgeons when compared to senior surgeons, and exhibited high reliability (ICC 0.962) (27). Fabijian explore open-source artificial intelligence models, including ChatGPT, Microsoft Bing, and Scholar A, in scoliosis classification. In comparing real and AI-generated scoliosis classifications, both demonstrated impeccable precision in all posturographic images, achieving total accuracy (1.0, MAE = 0.0) and remarkable inter-rater agreement, as evidenced by a perfect Fleiss' Kappa score. This high level of consistency is observed across scoliosis cases with Cobb's angles ranging from 11–92 degrees (12).

AI significantly enhances the efficiency of scoliosis image processing and classification. Traditionally, manual measurement of the Cobb angle and Lenke classification are time-intensive and prone to subjectivity. With your work, AI can automatically and accurately segment images and measure the Cobb angle, greatly improving diagnostic consistency and efficiency. This automated image processing supports standardized diagnosis and treatment planning for scoliosis. As multi-center data validation progresses, AI will offer a reliable basis for scoliosis image classification, likely becoming an essential diagnostic tool that optimizes clinical workflows.

##### 4.4.2.2 Blue cluster

This cluster mainly represents AI application in surgical treatment of scoliosis, AI technology significantly improves surgical precision, safety, and outcomes.

Intraoperative robots and navigation systems have demonstrated great potential. In 1992, Dr. Barger performed the first orthopedic surgery assisted robot (28). Currently, surgical robots are widely used in spinal surgery, especially in spinal deformity correction procedures. The robots are available in a range of designs, offering different levels of assistance that can be classified into three broad categories: (1) Supervisory-Controlled Systems: These systems are pre-programmed with specific actions, which the robot executes autonomously under the close supervision of a surgeon. (2) Telesurgical systems: Exemplified by the Da Vinci robot (Intuitive Surgical, Sunnyvale, California), these systems grant the surgeon full control over the robot's movements from a remote command station. (3) Shared-Control Models: In these systems, both the surgeon and the robot simultaneously control the motions, embodying a form of co-autonomy (29). Currently, the most commonly used spine robots are shared control system robots. The navigation system, combined with intraoperative imaging, can provide real-time stereoscopic images of the spine. By using feedback from registered trackers, it provides dynamic positioning to the surgeon. Both the robot and navigation system need to import the patient's spinal two-dimensional images into the workstation during surgery, generating three-dimensional images through computation.

Pedicle screw fixation is one of the most commonly used surgical techniques in spine surgery today. It holds significant value and importance for the reconstruction of the spine in patients with spine deformities (30). The assessment of pedicle screw placement accuracy has always been a persistent and widely focused concern for spine surgeons (31). Robotic navigation collects preoperative CT scan image data and uploads it to the system. Spinal surgeons can use the robotic navigation system to design the optimal path for pedicle screw placement in advance for each patient. Several meta-analysis have summarize intraoperative navigation results in more accurate pedicle screw placement compared to non-navigated techniques (32–35). Operative time is found to be approximately a half hour longer on average in navigated compared to non-navigated surgery (36).

Previous research report indicates that the use of navigation increases radiation exposure to patients while reducing radiation exposure to surgeons and the surgical team (37, 38). The stochastic effects of radiation can be magnified in children because of their longer lifespan. However, the results in radiation is not consistent. Li reports less exposure in 3D C-arm groups (4.85 ± .44 μSv) compared to free hand group (15.97E ± 2.35 μSv). Compared to free hand and other spine robotic, Mazor spine system is superior in exposure time and dosage (39). However, the radiation imparted by CT navigation is at least more predictable than fluoroscopy and may be less dependent on the operator (38).

AI enhances surgical precision and safety. AI-assisted robotic systems and navigation technologies enable accurate pedicle screw placement, reducing intraoperative variability. These systems also allow for personalized surgical path planning tailored to patients' unique anatomical structures, especially valuable in complex spinal deformity corrections. Studies have shown that AI-based navigation systems improve screw placement accuracy while reducing radiation exposure. As these technologies continue to advance, they promise to set new standards for scoliosis surgery globally, delivering safer and more effective treatments.

##### 4.4.2.3 Green cluster

This cluster primarily focuses on Using AI to predict postoperative complications and scoliosis development can greatly ensure outcomes and reduce rates of complications.

(**1**) **Scoliosis development prediction:** Deng developed a CNN prediction model for scoliosis progression based on time series analysis and general regression methods, achieving an accuracy of over 85% with its inference model (40). Chui employed a vertebral landmark extraction method and Feedforward Neural Network to predict scoliosis progression. The mean absolute error for the intervertebral angle progression is 1.5 degrees, and the Pearson correlation for the predicted Cobb angles is 0.86. The classification accuracy for Cobb angles (<15°, 15–25°, 25–35°, 35–45°, >45°) is 0.85, with a sensitivity of 0.65 and a specificity of 0.91. The FNN demonstrated superior accuracy, sensitivity, and specificity, thereby aiding in the development of tailored treatments for potential scoliosis progression (41).

**(2) Complication prediction:** Based on demographics, primary/revision status, use of three-column osteotomy, upper-most instrumented vertebra (UIV)/lower-most instrumented vertebra LIV levels and UIV implant type screws, hooks, number of levels fused, and baseline sagittal radiographs from 510 ADS patients with 2-year follow-up, Scheer developed a successful model (86% accuracy, 0.89 AUC) that can predict Proximal Junction Failure or clinically significant Proximal Junction kyphosis(PJK) (42). Peng develops SMOTE model with the great value (AUC = 0.944, accuracy = 0.909, and F1 score = 0.667) for predicting the individual risk of developing PJK after long instrumentation and fusion surgery in Lenke V AIS patients (43).

AI models like CNN and feedforward neural networks FNN have demonstrated notable accuracy in predicting postoperative complications and scoliosis progression. These models allow for early identification of postoperative risks, such as proximal junctional kyphosis, helping clinicians develop tailored intervention plans for high-risk patients. As larger, multi-center clinical datasets become available, these models are expected to become standard tools in postoperative management for scoliosis, improving patient outcomes and reducing complication rates through personalized treatment planning.

### 4.5 AI in scoliosis and beyond: a comparative growth across spine surgery and medical specialties

The rapid growth of AI applications in scoliosis has led to its widespread use in image analysis, deformity classification, surgical planning, and postoperative complication prediction. However, this growth is not unique to scoliosis; it reflects a broader trend across spine surgery. Significant advancements in AI have also been observed in other areas of spine surgery, such as spine tumor detection and the management of degenerative spinal diseases. For instance, AI assists in the diagnosis of spinal tumors by identifying tumor morphology and location through imaging data, enabling early detection and supporting personalized treatment decision-making (44–47). In the screening of osteoporotic vertebral compression fractures (OVCF), AI algorithms significantly improve sensitivity and specificity, particularly valuable in aging populations (48–50). The proliferation of AI supports real-time three-dimensional imaging and navigation for complex anatomical structures, progressively enabling precision surgery.

Similarly, AI is demonstrating rapid growth in other medical specialties. In neuroscience, AI analyzes large-scale brain imaging data, aiding in early diagnosis and disease progression prediction for neurodegenerative disorders such as Alzheimer's disease (51, 52). In ophthalmology, AI has made groundbreaking advances in screening for diabetic retinopathy and early detection of glaucoma, greatly enhancing identification efficiency and accuracy (53, 54). The rapid growth of AI applications in these fields underscores the broad applicability of AI in medical imaging analysis, structural pathology recognition, risk prediction, and decision support.

While AI applications in scoliosis have specific clinical characteristics, their growth trajectory aligns with that of other medical fields, driven by common technological advancements, namely, deep learning, computer vision, and natural language processing, along with the accumulation of clinical big data. The development of AI in scoliosis relies on the precision of image segmentation and quantitative measurement to support diagnosis and surgical planning. Additionally, the use of AI in predicting postoperative complications and designing personalized intervention plans showcases its value in managing complex cases. Thus, the growth of AI applications in scoliosis reflects both general progress in image analysis and real-time navigation and the unique requirements for managing spinal deformities.

### 4.6 Limitations

This study had several limitations. First, the data for the article is solely sourced from the Web of Science Core Collection database, excluding PubMed, Embase, Scopus, and other databases. Additionally, we only included studies published in English, which might have led to the omission of some relevant research. Second, According to our search criteria, we should include all types of scoliosis, including AIS and ADS. However, our analysis results primarily focuses on AIS and lacks analysis related to ADS. This may be due to the fact that most current AI research is concentrated on AIS. Third, because the database is continuously updated, some recently published high-quality papers may be underestimated. Fourth, only studies published in English are include, which may neglect studies in other English. Besides, only articles indexed in the Web of Science are included, which may have resulted in the omission of some literature. Nevertheless, the conclusions drawn in the article still hold significant reference value and are expected to bring new breakthroughs in the diagnosis and treatment of scoliosis using artificial intelligence.

## 5 Conclusion

This study compiled 718 publications covering AI in scoliosis and showed that the direction of these studies is likely in transition from cerebral palsy to machine learning and deep learning. It provides guidance for further research and clinical applications on AI application in scoliosis.
